# Putative regulatory functions of SNPs associated with bronchial asthma, arterial hypertension and their comorbid phenotype

**DOI:** 10.18699/VJ21.099

**Published:** 2021-12

**Authors:** I.A. Goncharova, E.Yu. Bragina, I.Zh. Zhalsanova, M.B. Freidin, M.S. Nazarenko

**Affiliations:** Research Institute of Medical Genetics, Tomsk National Research Medical Center of the Russian Academy of Sciences, Tomsk, Russia; Research Institute of Medical Genetics, Tomsk National Research Medical Center of the Russian Academy of Sciences, Tomsk, Russia; Research Institute of Medical Genetics, Tomsk National Research Medical Center of the Russian Academy of Sciences, Tomsk, Russia; Research Institute of Medical Genetics, Tomsk National Research Medical Center of the Russian Academy of Sciences, Tomsk, Russia; Research Institute of Medical Genetics, Tomsk National Research Medical Center of the Russian Academy of Sciences, Tomsk, Russia

**Keywords:** bronchial asthma, arterial hypertension, comorbidity, SNP, regulatory functions, бронхиальная астма, артериальная гипертензия, коморбидность, SNP, регуляторный потенциал

## Abstract

Linkage disequilibrium (LD) of single nucleotide polymorphisms (SNPs) of TLR4/AL160272.2 (rs1927914, rs1928298, rs7038716, rs7026297, rs7025144) was estimated in the Slavs of West Siberia. We further investigated an association of SNPs in TLR4/AL160272.2 (rs1927914, rs7038716, rs7025144), SERPINA1 (rs1980616), ATXN2/BRAP (rs11065987), IL2RB (rs2284033), NT5C2 (rs11191582), CARD8 (rs11669386), ANG/RNASE4 (rs1010461), and ABTB2/ САТ (rs2022318) genes with bronchial asthma (BA), arterial hypertension (AH) and their comorbidity. Then, the disease-associated SNPs were annotated in silico in relation to their potential regulatory functions. Strong LD was detected between rs1928298 and rs1927914, as well as rs7026297 and rs7038716 in the Slavs of West Siberia. It was found that the rs1927914 G allele of the TLR4 gene and the rs1980616 C allele of the SERPINA1 gene are associated with the predisposition to BA. These SNPs can affect binding affinity of transcription factors of the Pou and Klf4 families, as well as the expression levels of the TLR4 and SERPINA1 genes. The rs11065987 allele A of the ATXN2/BRAP genes, the rs11669386 A allele of the CARD8 gene, the rs2284033 allele G of the IL2RB gene, and the rs11191582 allele G of the NT5C2 gene were associated with the risk of AH. These variants can alter binding affinity of the Hoxa9, Irf, RORalpha1 and HMG-IY transcription factors, as well as the expression levels of the ALDH2, CARD8, NT5C2, ARL3, and SFXN2 genes in blood cells/vessels/heart, respectively. The risk of developing a comorbid phenotype of AD and AH is associated with the A allele of rs7038716 and the T allele of rs7025144 of the TLR4/AL160272.2 genes, the A allele of rs1010461 of the ANG gene and the C allele of rs2022318 of the ABTB2/CAT genes. Variants rs7038716 and rs7025144 can change the expression levels of the TLR4 gene in blood cells, while rs1010461 and rs2022318 influence the expression levels of the ANG and RNASE4 genes as well as the CAT and ABTB2 genes in blood cells, lungs/vessels/heart.

## Abstract (ru)

В работе оценено неравновесие по сцеплению однонуклеотидных вариантов (SNP) генов TLR4/AL160272.2 (rs1927914, rs1928298, rs7038716, rs7026297, rs7025144) у славян-жителей Западной Сибири. Проведен анализ ассоциаций SNP в области генов TLR4/AL160272.2 (rs1927914, rs7038716, rs7025144), SERPINA1 (rs1980616), ATXN2/BRAP (rs11065987), IL2RB (rs2284033), NT5C2 (rs11191582), CARD8 (rs11669386), ANG/RNASE4 (rs1010461) и ABTB2/САТ (rs2022318) при бронхиальной астме (БА), артериальной гипертензии (АГ) и их со- четании. Выполнено in silico аннотирование SNP, ассоциированных с данными заболеваниями, в отношении их регуляторного потенциала. В результате у славян-жителей Западной Сибири выявлено сильное неравно- весие по сцеплению rs1928298 и rs1927914, а также rs7026297 и rs7038716. Установлено, что к развитию БА предрасполагают аллель G rs1927914 гена TLR4 и аллель С rs1980616 гена SERPINA1. Данные SNP влияют на изменение аффинности транскрипционных факторов семейств Pou и Klf4, а также на экспрессию генов TLR4 и SERPINA1 в клетках крови соответственно. Аллель А rs11065987 генов ATXN2/BRAP, аллель А rs11669386 гена CARD8, аллель G rs2284033 гена IL2RB и аллель G rs11191582 гена NT5C2 ассоциированы с риском развития артериальной гипертензии. Данные варианты изменяют аффинность транскрипционных факторов Hoxa9, Irf, RORalpha1 и HMG-IY, а также экспрессию генов ALDH2, CARD8, NT5C2, ARL3 и SFXN2 в клетках крови, сосудах и сердце. Риск развития коморбидного фенотипа БА и АГ ассоциирован с аллелем А rs7038716 и аллелем Т rs7025144 генов TLR4/AL160272.2, аллелем А – rs1010461 гена ANG и аллелем С – rs2022318 генов ABTB2/САТ. Варианты rs7038716 и rs7025144 изменяют экспрессию гена TLR4 в клетках крови, а rs1010461 и rs2022318 – генов ANG и RNASE4, CAT и ABTB2 в клетках крови, легких, сосудов и сердца.

## Introduction

To date, association studies have identified a large number of genetic variants associated with the development of the risk of multifactorial diseases (https://www.ebi.ac.uk/gwas/). However, moving from establishing an association to understanding the mechanisms underlying diseases is a more difficult task. Linkage disequilibrium (LD) that differs in populations of different ethnic origin makes it difficult to identify causal genetic variants of a disease or a complex trait.

On the other hand, to understand the mechanisms of multifactorial diseases, it is important to assess the genetic variants’ functional significance, including annotation of the polymorphisms’ regulatory potential in relation to changes in the genes’ functional activity. Genetic variants affecting quantitative changes in gene expression profile (eQTL), or single nucleotide polymorphic variants (SNP), which have a regulatory status (rSNP) and are located in actively transcribed DNA regions, cause deviations from the optimal program of gene functioning in tissue cells and organs, which leads to an increased risk of diseases (van Arensbergen et al., 2019).

Bronchial asthma (BA) is a widespread heterogeneous disease characterized by chronic airway inflammation. Various comorbidities are common among BA patients, including allergic conditions: allergic rhinitis, dermatitis, and food allergy (Weatherburn et al., 2017), as well as some non-allergic pathological conditions: arterial hypertension (AH), obesity, type 2 diabetes mellitus, and other metabolic and endocrine disorders (Su et al., 2016). These diseases have common pathogenesis with BA and can modify clinical symptoms and the pathological process course in patients. For example, BA patients with concomitant AH, as a rule, have a number of phenotypic features, including older age, late-onset BA, high body mass index, and are characterized by a predominantly neutrophilic type of inflammation, which is not implemented through Th-2 lymphocytes (Moore et al., 2014).

evelopment of BA and AH comorbidity by analyzing the associative gene network structure and prioritization methods (Saik et al., 2018). As a result of the SNPs analysis of the selected priority genes it was shown that BA is associated with rs1928298 and rs1927914, localized at a distance of 19.6 and 1.7 Kb, respectively, from the 5′-end of TLR4 gene, as well as the intron variant rs1980616 of SERPINA1 gene. AH was associated with rs11065987 localized between ATXN2 and BRAP genes, as well as intron variants of IL2RB gene rs2284033, rs11191582 of NT5C2 gene, and rs11669386 of CARD8 gene. Rs7038716, rs7026297, and rs7025144 localized at the 1 intron of the AL160272.2 gene and at a distance of 35.6, 33.1, and 9.4 Kb, respectively, from the TLR4 gene 3′-end; rs1010461 (ANG/RNASE4), and intergenic rs2022318 localized between ABTB2 and CAT genes were associated with comorbid BA+AH phenotype (Bragina et al., 2018). The CAT and TLR4 genes’ haplotypes associations with the development of BA and the comorbid BA+AH phenotype have been established (Bragina et al., 2019). However, it has not previously been assessed which alleles of these SNPs are associated with diseases, what is the possible molecular mechanism explaining the obtained associations, and whether genetic variants have a regulatory potential for the functional activity of genes, which was the purpose of this study.

## Materials and methods

The study examined three groups of patients: with BA (n = 145, 73.1 % women, 25.9 % men, age 44.89 ± 8.86 years); with AH without BA in history (n = 144, 32.6 % women, 67.4 % men, age 51.27 ± 6.05 years); with BA and AH (n = 146, 72.6 % women, 27.4 % men, age 56.32 ± 10.47 years). The control group included individuals with normal blood pressure and no clinical asthma manifestations (n = 152, 73.7 % women, 26.3 % men, mean age in the group 47.75 ± 9.92 years). The diagnosis of “bronchial asthma” and “arterial hypertension” was established on the basis of patients’ clinical examination, according to generally accepted criteria. All individuals were Eastern Europeans (predominantly, Slavs).

Detailed methods for selecting and prioritizing SNPs, as well as the procedure for genotyping patients’ DNA samples using mass spectrometry on a Sequenom MassARRAY® device (USA), are given elsewhere (Bragina et al., 2018, 2019; Saik et al., 2018).

In this work, we evaluated linkage disequilibrium (LD) between single nucleotide polymorphisms (SNPs) of TLR4/ AL160272.2 genes (rs1927914, rs1928298, rs7038716, rs7026297, rs7025144) in Slavs living in West Siberia. Lewontin’s LD coefficient (D′) and Pearson’s correlation coefficient (r2) were estimated using HaploView v. 4.2.

Odds ratio (OR) and 95 % confidence intervals (CI) in relation to the risk of bronchial asthma (BA), arterial hypertension (AH), and their combination were calculated for SNPs in the region of TLR4/AL160272.2 (rs1927914, rs7038716, rs7025144), SERPINA1 (rs1980616), ATXN2/BRAP (rs11065987), IL2RB (rs2284033), NT5C2 (rs11191582), CARD8 (rs11669386), ANG/RNASE4 (rs1010461) and ABTB2/CAT (rs2022318) genes using logistic regression. Sex and age were used as covariates in the regression analysis. Odds ratios were calculated by exponentiating corresponding regression coefficients. For statistical analysis, Stats and Genetics packages were used in R program environment (The R Foundation).

To assess the SNPs regulatory potential associated with these diseases, the following databases were used: rSNPBase v. 3.1 (http://rsnp3.psych.ac.cn), HaploReg v. 4.1 (https://pubs.broadinstitute.org/mammals/haploreg/haploreg. php), RegulomeDB v. 2.0 (https://www.regulomedb.org/ regulome), GTEx Portal (https://gtexportal.org/home), Blood eQTL browser (https://genenetwork.nl/bloodeqtlbrowser). The search for data on the relationship of the studied genetic variants with diseases was carried out using the DisGeNET resource (https://www.disgenet.org/).

The study was carried out using the Biobank of the Population of Northern Eurasia on the basis of the Core Facilities Center of Scientific Research Equipment and Experimental Biological Material “Medical Genomics” of the Research Institute of Medical Genetics of the Tomsk National Research Medical Center of the Russian Academy of Sciences. The study protocol was approved by the Ethics Committee of the Research Institute of Medical Genetics (Protocol No. 2 dated 05/30/2016). Informed consent was obtained for all participants.

## Results and discussion

Linkage disequilibrium analysis of genetic variants located at locus 9q33.1 (TLR4/AL160272.2)

Since five of the associated variants – rs1927914, rs1928298, rs7038716, rs7026297, and rs7025144 – are located in the 9q33.1 locus (TLR4/AL160272.2), at the first stage we used all available individuals (n = 587) to analyze LD between the SNPs, which showed the presence of two blocks, one of which included rs1928298 and rs1927914 (D′ = 0.974; r2 = 0.949) (see the Figure).

**Fig.**
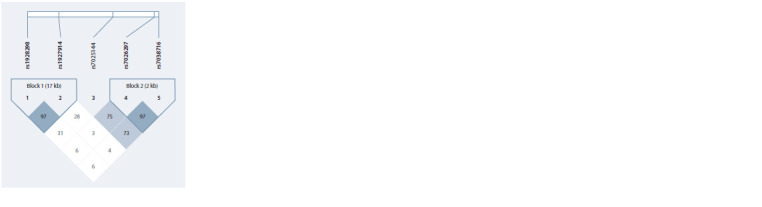
Haplotype structure of locus 9q33.1 (TLR4/AL160272.2), including rs1927914, rs1928298, rs7038716, rs7026297, and rs7025144 in the Slavs of West Siberia.

The haplotype structure of the 9q33.1 locus (TLR4/ AL160272.2) in the Slavs of West Siberia did not differ from that characteristic in the USA Caucasians (http://www. ensembl.org/Homo_sapiens/Variation/HighLD?db=core; r=9:117684048-117685048;v=rs1928298;vdb=variation; vf=729411740#373514_tablePanel/.

Association analysis of SNPs with bronchial asthma, arterial hypertension, and their comorbid phenotype

We chose variants in linkage equilibrium (rs1927914, rs7038716) and a SNP that showed weak LD with the second block (rs7025144) to carry out association analysis. As a result, we revealed an association between BA and the G allele of rs1927914 of TLR4 gene and allele C of rs1980616 of SERPINA1 gene; between AH and allele A of rs11065987 (intergenic region ATXN/BRAP), allele A of rs11669386 of CARD8 gene, allele G of rs2284033 of IL2RB gene, and allele G of rs11191582 of NT5C2 gene; and between combined pathology (BA+AH) and allele A of rs1010461 of ANG/RNASE4 genes, allele T of rs7038716 and allele T of rs7025144 of AL160272.2 gene, and allele C of rs2022318 (intergenic region ABTB2/CAT) (Table 1).

**Table 1.**
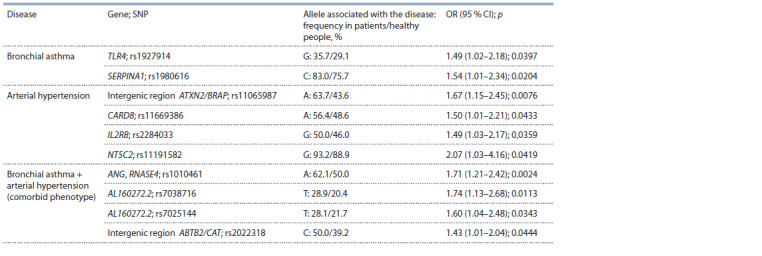
Genetic variants associated with the development of BA, AH and their combination

Assessment of the regulatory potential of SNPs associated with bronchial asthma

Regulatory regions of the genome are characterized by the presence of modified histones (methylated H3K4me1, H3K4me3 and acetylated H3K27ac, H3K9ac), which are the “labels” of active promoters and enhancers in various cells, including blood cells and cells in target organs for BA and AH – lungs, blood vessels, heart, and brain (Table 2). Thus, the variants rs1927914 and rs1980616 associated with BA are located in the promoter region of TLR4 gene and the enhancer of SERPINA1 gene in blood cells (monocytes). In addition, histone modifications in the rs1980616 (SERPINA1) region are recorded in other cells and tissues, including the lungs (see Table 2).

**Table 2.**
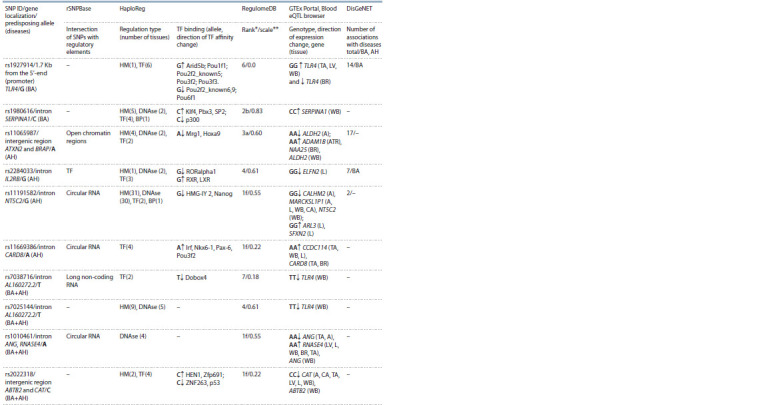
Regulatory potential of SNPs associated with BA, AH and their combination Notе. A – aorta; CA – coronary arteries; TA – tibial artery; LV – left ventricle; WB – whole blood; Br – brain; Atr – atrium; L – lungs; HM – histone modifications; DNAse – DNAse I-hypersensitive sites; TF – transcription factors; BP – bound proteins. * Ranking of the regulatory potential: 1f – eQTL + binding TF/DNAse I-hypersensitive site; 2b – eQTL + binding of TF + other regulatory elements + DNAse I-hypersensitive region; 3а – binding of TF + any TF motif + DNAse I-hypersensitive site; 4 – binding of TF + DNAse I-hypersensitive site; 6 – any TF motive; 7 – others. ** The closer the scale value is to one, the higher the SNP regulatory potential.

Genetic variants can alter the transcription factors (TF) affinity and regulated genes expression. It was shown that the G allele of rs1927914, located in TLR4 gene promoter, has a multidirectional effect on the binding of TFs of the Pou family (see Table 2). It is known that the Pou proteins, Oct-1 and Oct-2, activate the expression of interleukin-5 gene in the PER-117 cell line, by binding to the CLE0 promoter element (Thomas et al., 1999), which, in turn, causes airways inflammation and is considered as a target for BA therapy (Busse et al., 2019). In another study (Aneas et al., 2020), rs1888909 variant associated with BA and located at a distance of 2.3 Kb from the 5′-end of IL33 gene was found to be associated with the expression of this gene in epithelial cells in respiratory tract and the protein level in blood plasma, through the differential affinity of Oct-1 (Pou2f1) with a risk allele for this disease. It is possible that a similar mechanism is responsible for the association between the G allele of rs1927914 located 1.7 Kb upstream of TLR4 gene and BA.

The C allele of rs1980616 of SERPINA1 gene is associated with an increase in the affinity of TF Klf4 involved in inflammatory reactions, airway remodeling, and control of Th2-type cellular response (Tussiwand et al., 2015). In mice, in suppressor cells of myeloid origin and in epithelial cells of the respiratory tract, after exposure to the allergen, Klf4 gene expression increased in the direct proportion to the severity of allergic asthma (Nimpong et al., 2017). Among the other variants studied in this study, rs1980616 has the highest regulatory potential with the rank of 2b and the scale of 0.83 (see Table 2).

According to the GTEx Portal and Blood eQTL browser, all studied SNPs are eQTLs and affect nearby genes expression (cis-eQTL) as well as the expression of those located at a significant distance (trans-eQTL) in different tissues. According to the up-to-date information, rs1927914 and rs1980616 associated with BA are connected with changes in the expression of TLR4 and SERPINA1 genes, respectively. The GG genotype of rs1927914 is associated with an increase in the expression of TLR4 gene in whole blood, arteries, and left ventricular myocardium and with a decrease in the expression of this gene in brain tissues (cerebellum), and the CC genotype of rs1980616 is associated with an increase in the expression of SERPINA1 gene in whole blood (see Table 2).

The rs1927914 (TLR4) variant associated with BA is associated in this study with the severity of BA in Chinese population (Zhang et al., 2011). This variant is included in an LD of 13 rSNPs, of which rs2737190 is associated with chronic obstructive disease development and pulmonary tuberculosis with a multidirectional effect (https://www.disgenet.org/ browser/2/1/1/rs2737190/).

In the present work, the association of rs1980616 of SERPINA1 gene with the development of BA was established for the first time. Previous studies have shown that chromosomal region 14q23-q32 is associated with blood IgE levels, bronchial hyperreactivity, and other signs of allergic inflammation in different populations (Malerba et al., 2001). The role of the proteins coded by the serpin family genes in the pathogenesis of BA is unknown; however, they can exhibit a protective effect by inhibiting endogenous proteases associated with inflammatory response, which merits further investigation.

Assessment of the regulatory potential of SNPs associated with arterial hypertension

Histone proteins modifications for the rs2284033 variant localized in an intron of IL2RB gene are recorded in peripheral blood leukocytes (T and NK cells) and for rs11191582 localized in an intron of NT5C2 gene in a wide range of cells and tissues, including blood cells, lungs, vessels, heart, brain (see Table 2).

Single nucleotide variants associated with AH affect the change in TF affinity. Allele A of rs11065987 localized in the intergenic region ATXN2/BRAP is associated with a decrease in the TF Mrg1 and Hoxa9 binding affinity (see Table 2). Previously, in patients with hypertension, peripheral blood CD34+ hematopoietic stem cells showed a decrease in HOXA9 gene expression, which may be associated with a decrease in circulating endothelial progenitor cells and impaired neovascularization and vascular damage repair (Pirro et al., 2007).

Allele A of the intron variant rs11669386 of the CARD8 gene affects the increase in the affinity of several TFs, including factors of the Irf and Pou3f2 families (see Table 2). In experimental studies of model animals, it was shown that the regulatory factor interferon 1 (Irf) plays a leading role in the regulation of cardiac remodeling during pressure overload (Jiang et al., 2014).

Allele G of rs2284033 of IL2RB gene affects a decrease in affinity for TF RORalpha1 and an increase in RXR/LXR (see Table 2). No association of RORalpha1 and RXR/LXR with hypertension was found in the available scientific literature; however, it was shown that adenovirus-mediated overexpression of RORalpha1 suppresses TNF-alpha-induced expression of adhesion molecules VCAM-1 and ICAM-1 in umbilical vein endothelial cells (Migita et al., 2004). LXR/RXR family transcription factors regulate inflammation, cholesterol homeostasis, lipid, and glucose metabolism. By modulating the components of the renin-angiotensin-aldosterone system LXR/RXR, they reduce peripheral vascular resistance and blood pressure (Cannon et al., 2016).

Allele G of rs11191582 of NT5C2 gene is associated with a decrease in affinity for the transcription factors HMG-IY and Nanog (see Table 2). Previously, it was shown that HMG-IY is involved in the chromatin structure formation and regulation of transcription of many genes. Changes in HMG-IY levels have been shown to affect promoter activity and IL2 gene expression in human cell lines (Himes et al., 2000). An important role of T-cells has been shown in the pathogenesis of hypertension, namely the activation of Th1 lymphocytes which are producers of IL2. Increased production of IL2 along with the cytokines IL1β, IL6, TNFα, and IFNγ promotes vascular inflammation and hypertension development (Schiffrin, 2014).

The genetic variants associated with hypertension are eOTLs. It was shown that rs11065987 (ATXN2/BRAP) is a cis- and trans-eQTL and affects the ALDH2, ADAM1B, and NAA25 genes activity in various cells and tissues (see Table 2). The AA genotype of rs11065987 (ATXN2/BRAP) is associated with a decrease in ALDH2 gene expression in aorta and an increase in its expression in whole blood leukocytes, as well as with an increase in ADAM1B gene expression in heart atrium and NAA25 gene in basal ganglia. Previously, a link with the cardiovascular disease and hypertension for some of these genes and their protein products was established. Thus, BRAP gene variants were found to be associated with the risk of carotid atherosclerosis (Liao et al., 2011); protein products of genes ATXN2 and SH2B3 were involved in AH development (Siedlinski et al., 2020); rs671 of ALDH2 gene was associated with a decrease in the risk of AH development (Mei et al., 2020). For the variant rs11065987, an association with many pathological conditions was registered, including the level of lipids in blood (Willer et al., 2013), body mass index (Locke et al., 2015), ischemic stroke, ischemic heart disease (Dichgans et al., 2014) and AH (Levy et al., 2009).

The rs 11669386 variant is associated with CARD8 gene functional activity, its SNPs are significant for the formation of susceptibility to hypertension, aortic aneurysm, and stroke (Zhao et al., 2016). The AA genotype of rs11669386 is associated with an increase in CARD8 gene expression in arteries and brain, as well as with an increase in CCDC114 gene expression in tibial artery, lungs, and whole blood (see Table 2). Proteins CARD8 and NLRP3 together control the activity of inflammatory caspase-1 and are involved in the regulation of caspase-1-mediated activation of IL1B in mouse macrophages, dendritic cells, and macrophages of human peripheral blood (Abdelaziz et al., 2015). As shown in a Ka-wasaki disease murine model, caspase-1 and IL-1B are important inflammatory cytokines in coronary artery disease development (Lee et al., 2012).

The rs2284033 variant has a pronounced effect on IL2RB gene expression in peripheral blood leukocytes (Westra et al., 2013), and the GG genotype is associated with a decrease in ELFN2 gene expression in lung tissues (see Table 2). Earlier, the association of rs2284033 with BA in total was established, which, however, diminishes when considering individual phenotypes associated with the disease age of onset (Moffatt et al., 2010). In addition, this SNP is in LD with 10 rSNPs, one of which (rs228953) has been shown to be associated with eosinophil counts in blood and the risk of BA (Han et al., 2020).

In our study, the G allele of rs2284033 (IL2RB) is associated with AH (see Table 1). As mentioned above, the role of immune system and inflammation in AH pathogenesis is currently well established. Increased expression of IL2RB gene was detected in leukocytes in patients with AH (Huan et al., 2015). In this regard, IL2RB gene polymorphism may predispose, in combination with other factors, to the development of AH.

The GG genotype of rs11191582 (NT5C2) is associated with a decrease in CALHM2 and MARCKSL1P1 genes expression in aorta, NT5C2 and MARCKSL1P1 genes in whole blood cells, and an increase in ARL3 and SFXN2 genes expression in the lung tissue (see Table 2). The protein product of CALHM2 gene modulates calcium homeostasis, the maintenance of which is necessary for the performance of cellular functions, such as contraction, proliferation, migration, and growth, the disruption of which is associated with the development of cardiovascular diseases. It was previously shown that rs11191582 is localized in the same LD block with rSNPs (rs11191548, rs11191559, rs11191580, rs11191593, rs12413409, and rs943037), for which a relationship with blood pressure regulation and coronary artery disease development was established (Matsunaga et al., 2020). In the nucleotide sequence of ARL3 gene, SNPs were identified that are located in different LD blocks and were associated with blood pressure level. This suggested that in the 10q24.32 locus, where ARL3 and SFXN2 genes are located, numerous “causal” genetic variants of susceptibility to AH may be situated (Li et al., 2017).

Assessment of the regulatory potential of SNPs associated with a comorbid phenotype – BA and AH

Genetic variants associated with BA + AH phenotype are located in DNAse I hypersensitive sites (rs1010461, rs7025144), in the enhancer region (rs7025144, rs2022318) and affect TF affinity (rs7038716, rs2022318) (see Table 2).

The rs1010461 variant localized in intron region of ANG and RNASE4 genes does not change the TF affinity, but its relationships with the functional activity of these genes in blood cells, arteries, heart, lungs, and brain have been shown (see Table 2). It was demonstrated that the AA rs1010461 genotype is associated with a decrease in ANG gene expression in tibial artery and aorta, but an increase in the expression level of this gene in whole blood cells. The AA genotype is associated with an increase in the expression level of ribonuclease 4 (RNASE4) gene in whole blood leukocytes, tissues of the left ventricle, lungs, brain, and tibial artery

RNASE4 gene has the same promoter and is co-expressed with angiogenin gene (ANG). However, the role of RNASE4 in the development of cardiovascular and bronchopulmonary diseases is unknown. In turn, angiogenin is a potent inducer of blood vessel formation and, possibly, can be used as a serum marker for the development of cardiovascular diseases (Yu et al., 2018). During the period of an asthmatic attack, an increase in vascular endothelial growth factor and angiogenin is recorded in the patients’ sputum, which is significantly reduced after corticosteroid therapy (Abdel-Rahman et al., 2006). It is possible that the treatment of asthma with corticosteroids can act as an unfavorable factor for the development of subsequent arterial hypertension in individuals predisposed to this pathology.

Allele T of rs7038716 (AL160272.2) is associated with a decrease in affinity for TF Dobox4. Genotypes TT rs7038716 and TT rs7025144 are associated with a decrease in TLR4 gene functional activity in whole blood leukocytes (see Table 2). In the present study, BA is associated with alleles and genotypes associated with an increase in functional activity of TLR4 gene, while BA and AH comorbidity is associated with genotypes characterized by a decreased TLR4 gene expression in blood cells. This result requires further research and explanation

Allele C rs2022318 (ABTB2/CAT ) is associate p53. P53 expression is increased with an increase in affinity for TF HEN1 and Zfp691, but with a decrease in affinity for ZNF263 and

ased in response to DNA damage, hypoxia, and oxidative stress, which provides cellular protection. New evidence is emerging to support the protective effect of p53 in inflammatory processes in the lungs. In particular, it has been shown that p53 deficiency in vascular lung endothelial cells is associated with severe respiratory disorders (Uddin, Barabutis, 2020). For ZNF263, an association with atherosclerosis was shown through the regulation of TGFB1 gene expression, the protein product of which is actively involved in various diseases’ pathogenesis (Dhaouadi et al., 2014).

The CC genotype rs2022318 is associated with a decrease in the expression of ABTB2 gene in whole blood cells and catalase gene (CAT) in tissues of aorta, left ventricle, lungs, coronary arteries, and whole blood cells (see Table 2). Catalase is an important antioxidant enzyme the decreased activity of which is seen in many diseases associated with oxidative stress. It has been shown that SNPs localized in the catalase gene promoter are associated with the development of cardiometabolic diseases (Doğan et al., 2019) and bronchial asthma (Taniguchi et al., 2014). There are no data on the relationship of ABTB2 gene polymorphism, its functional activity, or protein product with AH and BA in the available scientific literature.

## Conclusion

Thus, SNPs associated with the studied phenotypes – BA, AH, and BA+AH, are regulatory (rSNP), localized in actively transcribed regions of the genome, are eQTLs, and affect functional activity of various genes in blood cells and target organs of the diseases – lungs, blood vessels, heart, and brain.

The relationship of rs1927914 and rs1980616 with BA can be explained by: the effect of the G allele rs1927914 on the change in the affinity of transcription factors of the Pou family and the relationship of the GG genotype with an increase in the level of TLR4 gene expression in blood cells; the relationship of the C allele rs1980616 with an increase in the affinity of the transcription factor Klf4, which is involved in inflammation, remodeling of the airways and control of Th2-type cell response, as well as the association of the CC genotype with increased expression of the SERPINA1 gene in blood cells.

The association of rs11065987, rs2284033, rs11191582, and rs11669386 with AH may be based on the following: the effect of the A allele rs11065987 on a decrease in the affinity of the Hoxa9 transcription factor and a decrease in the pool of endothelial progenitor cells, which disrupts neovascularization and changes in ALDH2 expression of vascular damage, and in blood cells and blood vessels; the relationship of the A allele rs11669386 with an increase in the affinity of the transcription factor Irf, which plays a leading role in the regulation of cardiac remodeling under pressure overload, and the Pou3f2 factor associated with the development of left ventricular remodeling in hypertension, the AA genotype is associated with an increase in CARD8 gene expression in blood vessels, the protein product of which is included in the composition of the inflamosome and is involved in the regulation of inflammatory reactions in various diseases, including cardiovascular ones; the effect of the G rs2284033 allele on a decrease in affinity for TF RORalpha1, which suppresses TNF-alpha-induced expression of adhesion molecules VCAM-1 and ICAM-1 in endothelial cells and the relationship of the GG rs11191582 genotype with a change in the level of expression of NT5C2, ARL3, and SFXN2 genes in blood/vascular cells and heart.

The association of rs7038716, rs7025144, rs1010461, and rs2022318 with the comorbid phenotype of BA and AH may be due to: the relationship of the TT and TT genotypes rs7038716 and rs7025144 with a decrease in TLR4 gene functional activity in blood cells; the relationship of the AA rs1010461 genotype with a change in ANG gene expression in blood and vascular cells, the protein product of which plays an important role in the pathogenesis of both AH and BA; the influence of the C allele rs2022318 on the increase in the affinity of the transcription factor p53, the expression of which increases during inflammatory processes in the lungs and the connection of the CC genotype with a decrease in CAT gene expression in blood cells, lungs, blood vessels and heart, the decrease in the activity of which is observed in diseases associated with oxidative stress.

## Conflict of interest

The authors declare no conflict of interest.
